# Evaluating the Reliability and Validity of Predictive Anthropometric Equations for Estimating Fat Mass, Lean Mass and the Role of Maturity Offset in Lean Mass Prediction Within Professional, Academy Soccer Players from the United Kingdom

**DOI:** 10.3390/sports14030091

**Published:** 2026-03-02

**Authors:** Elena Efstathiou, Laura J. Wilson, Brent Dickinson, Christopher Curtis

**Affiliations:** 1 Watford Football Club, Vicarage Road, Watford WD18 0ER, UK; eefstathiou@brentfordfc.com (E.E.); brent.dickinson@watfordfc.com (B.D.); 2 School of Life Sciences, University of Westminster, London W1W 6UW, UK; 3 London Sport Institute, Middlesex University, London NW4 4BT, UK; l.wilson@mdx.ac.uk; 4 Physical Activity, Physical Education, Health and Sport (PAPESH) Research Centre, Faculty of Sport Science, Reykjavik University, 102 Reykjavik, Iceland

**Keywords:** soccer, body composition, growth, maturation, athlete development

## Abstract

The reliability and validity of anthropometric equations remain uncertain in young athletes experiencing biological maturation. This study assessed the reliability and validity of anthropometric equations against dual-energy X-ray absorptiometry (DXA)-derived fat mass (FM) and lean mass (LM) values and examined the influence of maturity offset within academy soccer players. Twenty-five male academy soccer players (age: 18.6 ± 0.8 years, height: 182.7 cm ± 5.9 cm, BM: 79.3 kg ± 7.6 kg) completed skinfold and DXA assessments. FM and LM were estimated using commonly adopted anthropometric equations. Reliability and validity were assessed. Linear regression examined the influence of maturity offset. Acceptable agreement for the equations of Wilmore & Behnke and Oliver et al. for LM and FM was observed (FM; ICC: 0.858–0.891, CV%: 8.1–8.8 ± 4.6–6.4, LoA: 2.62–3.06 to −1.33–−1.62, *ES*: 0.27–0.47, *Z* = −2.257–−3.150; LM: ICC: 0.886–0.905, CV%: 2.9–3.3 ± 1.3, LoA: 5.17–5.62 to 0.54–0.78, *ES*: 0.42–0.48, both *p* < 0.001). Bland–Altman inspection showed mean bias and wide LoA for all equations. Maturity offset modestly predicted LM for all equations. Observed anthropometric equations have limited validity vs. DXA-derived FM and LM in academy soccer players. Maturity offset warrants consideration for maturity-sensitive, population-specific equations to avoid systematic errors.

## 1. Introduction

Anthropometric and body composition variables are routinely assessed by sport science practitioners to provide an indication of an athlete’s fitness and health, often in response to changes in nutritional or training status [1,2]. Given the practicalities within applied sport settings, body composition is normally estimated using a two-compartment model (e.g., skinfolds, SKF; bioelectrical impedance, BIA) or a three-compartment model (e.g., BIA; dual-energy X-ray absorptiometry, DXA) to calculate fat-free mass (FFM), fat mass (FM), lean mass (LM), and, in the case of DXA, bone mineral content (BMC) [2,3,4]. Despite being susceptible to errors, SKF measurements are widely considered a reliable and feasible approach for monitoring longitudinal changes in body composition and evaluating the effectiveness of nutritional and training strategies, provided they are conducted by trained anthropometrists following standardized protocols [4,5]. Similarly, DXA is widely regarded as a reference method for body composition assessment, offering superior accuracy, precision, and reproducibility compared to other methods [6,7,8]. DXA can indicate the magnitude of FFM, FM, LM, and BMC changes over time and offers reliable segmental data; however, its application in applied sport settings is often limited by issues of accessibility, cost, and the requirement for specialized equipment and trained personnel [9].

Specific to soccer, the measurement of FM and LM within players has been used to monitor performance and adaptation to training and match-play scenarios, with an excess of FM shown to impair a number of soccer-specific performance parameters, including (but not limited to) aerobic capacity, power-to-weight ratio, repeated sprint ability, and flexibility [10,11,12]. In the case of LM, it is viewed as a fundamental determinant of strength, power, and injury resilience in team sport athletes, whereby training and match-play demands are underpinned by explosive movements and high-intensity actions [13]. Within youth soccer, many clubs have developed academy systems to support the development of age category players [14]. As part of these systems, academy sport science and medicine departments may be required to deliver integrated sport science and medical services to optimize player development and progression, part of which may involve body composition testing and monitoring as part of Long-Term Athletic Development (LTAD) models [15,16,17]. From a physical perspective, as soccer players transition through an academy pathway, they undergo distinct stages of biological maturation [18,19]. Whilst acknowledging inter-individual variability in the timing of peak height velocity (PHV), it has been reported that under-18 (U18) academy soccer players exhibit significantly lower body mass (BM) and LM than both under-21 (U21) and first-team counterparts, despite no differences in stature [20,21].

Anthropometric predictive equations to measure FM and FFM in both soccer [8,22,23,24] and futsal [25] have shown mixed results regarding their validity when compared against DXA-derived FM and FFM values within these cohorts. Anthropometric predictive equations often assume homogeneity within age groups, overlooking inter-individual differences in biological maturation [26], which, from an applied perspective, may lead to over- or underestimation of FFM and LM values and may not accurately reflect the physiological profiles of trained youth athletes, thereby potentially limiting their use in academy soccer players. The consideration of maturity offset was developed by Mirwald et al. [27] using anthropometric measurements and chronological age to estimate years from PHV and may be a key factor in determining the accuracy of anthropometric predictive equations within academy-age athletes, with maturity offset shown to significantly influence muscle mass, skeletal development, and overall body composition [28]. Given the commonality of body composition monitoring within soccer academies, it is imperative that sport science practitioners utilize appropriate screening methods and (where applicable) anthropometric predictive equations, to enhance screening precision within LTAD models. To date, few studies have specifically examined the reliability and validity of anthropometric predictive equations within academy soccer players when factoring in the influence of biological maturation. With this in mind, the aims of the present study were to (a) assess the reliability and validity of anthropometric predictive equations against DXA-derived FM and LM values and (b) examine the influence of maturity offset on the LM values estimated by specific anthropometric predictive equations within professional academy soccer players from the United Kingdom (UK).

## 2. Materials and Methods

The present investigation employed an observational, cross-sectional design, and was conducted and reported in accordance with the Strengthening the Reporting of Observational Studies in Epidemiology (STROBE) guidelines [29]. A convenience sample was drawn from the U18 and U21 playing squads of a professional, academy soccer team based in the United Kingdom. All procedures were reviewed and approved by the University of Westminster and Middlesex University Research Ethics Committees (approval codes: University of Westminster: ETH2425-1948/05-2025, Middlesex University: ETH2425-0227), in accordance with the 1964 Declaration of Helsinki for research involving human participants. This study utilized a definitive sample size comprising all eligible participants within the football club’s U18 and U21 playing squads; therefore, post hoc power was calculated after data collection. The single assessment time point occurred during the playing squad’s respective testing week at the start of the 2025/26 pre-season period. All participants were assessed using DXA and anthropometry.

### 2.1. Subjects

A total of 25 professional male academy soccer players from a UK-based Championship football club voluntarily participated in the study (age: 18.6 ± 0.8 years, height: 182.7 cm ± 5.9 cm, BM: 79.3 kg ± 7.6 kg). The Championship represents the second-highest tier of competition within English soccer. During the off-season, players followed a prescribed three-day-per-week fitness program and received generalized dietary guidance from the football club’s academy performance nutritionist. These recommendations included moderating dietary intakes of approximately <4 g·kg·BM^−1^ of carbohydrate, 20–35% of total energy from fat and protein intakes of 1.6–2.2 g·kg·BM^−1^. Compliance was monitored to ensure the preservation of physical qualities during the off-season period. Inclusion criteria were as follows: (i) current registration with the soccer club’s U18 or U21 squad, (ii) absence of fever or illness at the time of assessment, and (iii) no recorded injury during the study period. Following a detailed explanation of the study procedures, informed consent was obtained from all participants.

### 2.2. Procedures

*Skinfolds*: Participants arrived at the training ground at 09:00 in a rested state, having completed an overnight fast. Standing height, sitting height and BM were measured using a stadiometer (Seca 213, Hamburg, Germany) to the nearest 1 mm and a calibrated digital scale (Seca 875, Hamburg, Germany) to the nearest 0.1 kg, respectively. Skinfold thicknesses were measured at ten anatomical sites. Eight skinfold sites were measured in accordance with the International Society for the Advancement of Kinanthropometry (ISAK) standardized procedures [30] which comprised biceps, triceps, subscapular, suprailiac, supraspinale, abdominal, anterior thigh, and medial calf. Additionally, two additional measures were collected at the chest and midaxillary to satisfy the requirements of specific prediction equations used to measure FM. All measurements were taken by the football club’s academy performance nutritionist (a Level 1 accredited ISAK anthropometrist). Measurements were taken to the nearest 0.1 mm using skinfold calipers (Harpenden, Bowers Group Ltd., Burgess Hill, UK). FM was estimated using anthropometric equations that have been previously validated in male academy soccer players [8,22,23,24]. The equations applied were as follows:

- Durnin and Womersley 1974 [31] (9): *Fat mass (%) = (495/(1.1631 − 0.0632 × log_10_ (Biceps + Triceps + Subscapular + Suprailiac))) − 450*
- Slaughter et al. 1988 [32]: *If sum ≤ 35 mm: Fat mass (%) = 1.21 × (Triceps + Subscapular) − 0.008 × (Triceps + Subscapular)^2^ − 5.5. If sum > 35 mm: Fat mass (%) = 0.783 × (Triceps + Subscapular) + 1.6*
- Withers et al. 1987 [33]: *Fat mass (%) = (495/(1.0988 − 0.0004 × (Σ7SKF))) − 450. Where Σ7SKF = Triceps + Biceps + Subscapular + Supraspinale + Abdominal + Anterior Thigh + Medial Calf*
- Wilmore and Behnke 1969 [34]: *Fat mass (%) = (495/(1.08543 − 0.000886 × Abdominal − 0.0004 × Thigh)) − 450*
- Oliver et al. 2012 [35]: *Fat mass (%) = 0.132 × (Σ7SKF) + 3.530. Where Σ7SKF = Chest + Triceps + Subscapular + Midaxillary + Suprailiac + Abdominal + Thigh*

Once predictive FM was calculated by each anthropometric equation, the following equation was applied to each of the predictive anthropometric equations to provide an estimate of LM for each participant:

*Body mass (kg) − (FM (kg)/100) × Body Mass (kg)*

*Maturity Offset Calculation*: As per the methods of Mirwald et al. [27], the following formula was applied to calculate maturity offset for each participant:

*Maturity offset = −9.236 + (0.0002708 × leg length × sitting height) − (0.001663 × age × leg length) + (0.007216 × age × sitting height) + (0.02292 × weight/height ratio × 100).*

Participants were then classified into four maturity offset groups: 1–2 years post-PHV, 2–3 years post-PHV, 3–4 years post-PHV, and 4+ years post-PHV. This grouping enabled comparative analysis of LM estimation across different stages of maturation, recognizing that physical and physiological development continues beyond PHV [36].

*Dual-Energy X-ray Absorptiometry*: Players departed the football club’s training ground at ~13:30 for a staggered arrival at the laboratory. Individual DXA timeslots had been pre-allocated. Although DXA assessments are ideally performed after an overnight fast [37], afternoon scheduling was required to accommodate the players’ morning pre-season training commitments. To minimize fluctuations in LM values, and in accordance with existing recommendations [37], players consumed lunch at ~12.00–13.00 and refrained from further food intake for at least two hours prior to their individual scanning appointment. All players followed the same standardized protocol. Prior to scanning, the DXA scanner (Lunar iDXA, GE Medical Systems, Lunar Madison, WI, USA) was calibrated in accordance with manufacturer instructions. Participants were screened for any existing injuries or conditions that could contraindicate DXA assessment. Standing height and body mass were recorded and entered into the DXA system to initialize participant profiles, with all scans performed by the same qualified technician. LM was assessed using DXA, which operates based on differential tissue absorption of two X-ray energy peaks [6], and was analyzed using enCORE software (version 18, 2021). During the DXA procedure, participants were exposed to low levels of ionizing radiation (3.0 μGy per full-body scan), representing minimal health risk and comparable to daily environmental exposure over a 24 h period at sea level [37,38]. Participants were positioned supine on the scanning bed with hands pronated and placed by their sides, in accordance with manufacturer guidelines. To optimize scan accuracy, minimal clothing was worn, following the standardized procedures outlined by Nana et al. [7].

*Statistical Analysis*: Data were analyzed using the Statistical Package for the Social Sciences (SPSS Version 29.0; IBM Corp., Armonk, NY, USA). Normality was assessed via the Shapiro-Wilk test. Intra-observer reliability of skinfold measurements was assessed using ICC, technical error of measurement (TEM) and CV%, with results interpreted against the accepted TEM 5% threshold, as per the methods of ISAK [30,39]. Paired *t*-tests assessed predictive anthropometric equations and DXA for FM and LM. If the data was not normally distributed, a Wilcoxon Signed Ranks test was used. Intra-class correlation coefficients (ICC: two-way mixed method and absolute agreement) were used to assess the agreement between methods. Coefficients of variation (CV%; typical error expressed as a percentage of the subject’s mean score) and limits of agreement (LoA; mean bias ± 1.96 standard deviation: SD) were calculated and, provided that no significant differences existed, variables were deemed to have acceptable agreement if CV% was ≤10% and ICC was ≥0.8 [40]. Bland–Altman scatterplots were produced to evaluate potential bias between mean differences [41]. Linear regression analysis was conducted to determine the contribution of maturity group to predicted LM via the anthropometric equations. Effect sizes (*ES*) were calculated in accordance with Cohen’s d *ES* principles (0 < *ES* < 0.2: trivial; 0.2 < *ES* < 0.5: small; 0.5 < *ES* < 0.8: medium; and >0.8: large) [42]. An alpha level of *p* ≤ 0.05 denoted significance.

## 3. Results

All 25 players completed both the skinfold and DXA scan assessments. Post hoc analyses using the means of DXA-derived FM versus FM estimated via the predictive equation of Durnin and Wormsley, 1974 [31], with beta set at 0.80 and α being equal to 0.05 (two-tailed), yielded a statistical power of 0.95. No players recorded skinfold sums < 35 mm during their skinfold assessment; therefore, the Slaughter et al., 1988 [32], part of the predictive equation calculating FM based upon this (i.e., skinfold sums < 35 mm) was not used during analysis. Mean data for whole-body FM and LM are presented in Table 1 and Table 2, respectively. Intra-rater reliability of skinfold measurements demonstrated good reliability and was within acceptable TEM thresholds across all sites (ICC: 0.998, TEM: 3.74%, CV%: 0.92–1.93%, *p* < 0.001).

### 3.1. Fat Mass: Predictive Anthropometric Equations vs. Dual-Energy X-Ray Absorptiometry

**Figure 1:**
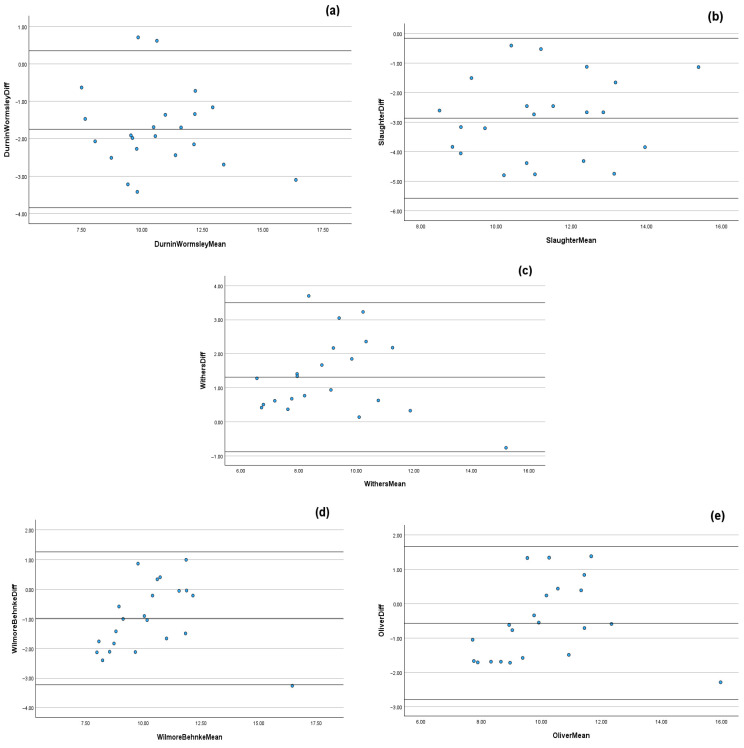
Bland–Altman scatterplots with limits of agreement (mean bias ± 1.96 SD) illustrating proportional bias at higher values for fat mass between dual-energy X-ray absorptiometry and the predictive anthropometric equations of Durnin and Womersley (1974) [31] (**a**), Slaughter et al. (1988) [32] (**b**), Withers et al. (1987) [33] (**c**), Wilmore and Behnke (1969) [34] (**d**) and Oliver et al. (2012) [35] (**e**).

Acceptable agreement was observed between DXA and the Wilmore & Behnke, 1969 [34] and Oliver et al., 2012 [35] anthropometric predictive equations (Wilmore & Behnke, 1969: ICC: 0.858, CV%: 8.8 ± 6.4, LoA: 3.06 to −1.33, *ES*: 0.47, *Z* = −3.150; Oliver et al., 2012: ICC: 0.891, CV%: 8.1 ± 4.6, LoA: 2.62 to −1.62, *ES*: 0.27, *Z* = −2.257, both *p* < 0.001; Table 1). Unacceptable agreement was observed between DXA and the Durnin & Womersley, 1974 [31], Slaughter et al., 1988 [32] and Withers et al., 1987 [33] anthropometric predictive equations (Durnin & Womersley, 1974: ICC: 0.793, CV%: 12.6 ± 5.9, LoA: 3.81 to −0.73, *ES*: 0.78; Slaughter et al., 1988: ICC: 0.531, CV%: 18.6 ± 9.6, LoA: 5.66 to −0.63, *ES*: 1.50; Withers et al., 1987: ICC = 0.839, CV%: 10.8 ± 7.8, LoA: 1.06 to −3.37, *ES*: 0.70; *Z* = −3.815, all *p* < 0.001; Table 1). Visual representation of the Bland–Altman scatterplots suggest notable mean bias for all predictive anthropometric equations, with wide limits of agreement, indicating substantial variability when measuring FM (Figure 1).

### 3.2. Lean Mass: Predictive Anthropometric Equations vs. Dual-Energy X-Ray Absorptiometry

**Figure 2:**
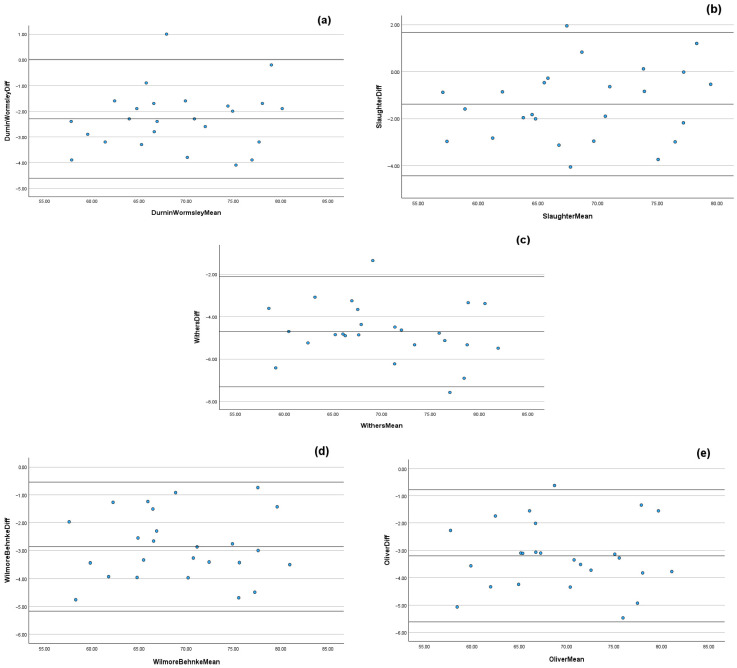
Bland–Altman scatterplots with limits of agreement (mean bias ± 1.96 SD) illustrating proportional bias at higher values for lean mass between dual-energy X-ray absorptiometry and the predictive anthropometric equations of Durnin and Womersley (1974) [31] (**a**), Slaughter et al. (1988) [32] (**b**), Withers et al. (1987) [33] (**c**), Wilmore and Behnke (1969) [34] (**d**) and Oliver et al. (2012) [35] (**e**).

Acceptable agreement was observed between DXA and the anthropometric predictive equations of Durnin & Womersley, 1974 [31], Slaughter et al., 1988 [32], Wilmore & Behnke, 1969 [34], and Oliver et al., 2012 [35] (Durnin & Womersley, 1974: ICC: 0.928, CV%: 2.5 ± 1.1, LoA: 4.62 to −0.03, *ES*: 0.35; Slaughter et al., 1988: ICC: 0.951, CV%: 1.8% ± 1.2, LoA: 4.44 to −1.67, *ES*: 0.21; Wilmore & Behnke, 1969: ICC: 0.905, CV%: 2.9 ± 1.3, LoA: 5.17 to 0.54, *ES*: 0.42; Oliver et al., 2012: ICC: 0.886, CV%: 3.3 ± 1.3, LoA: 5.62 to 0.78, *ES*: 0.48, all *p* < 0.001; Table 1). Unacceptable agreement between DXA and the Withers et al. 1987 [33] equation was observed (Withers et al. 1987: ICC = 0.797, CV%: 4.8 ± 1.3, LoA: 7.31 to 2.10, *ES*: 0.69, *p* < 0.001; Table 2). Visual representation of the Bland–Altman scatterplots suggest that, for all anthropometric predictive equations, there was notable mean bias, with wide limits of agreement indicating substantial variability when measuring LM (Figure 2). Linear regression demonstrated that maturity offset modestly predicted LM for all anthropometric predictive equations (Durnin & Womersley, 1974 [31]: R2_adj_ = 0.363, F_(1,23)_ = 14.66; Slaughter et al. 1988 [32]: R2_adj_ = 0.358, F_(1,23)_ = 14.37; Withers et al. 1987 [33]: R2_adj_ = 0.366, F_(1,23)_ = 14.85; Wilmore & Behnke, 1969 [34]: R2_adj_ = 0.389, F_(1,23)_ = 16.29; Oliver et al. 2012 [35]: R2_adj_ = 0.379, F_(1,23)_ = 15.65, all *p* < 0.001).

## 4. Discussion

The aims of the present study were to (a) assess the reliability and validity of anthropometric predictive equations against DXA-derived FM and LM values and (b) examine the influence of maturity offset on LM values estimated by each anthropometric predictive equation within professional, academy soccer players from the UK. When considering FM, acceptable agreement (ICC: >0.8 and CV%: <10%) was observed between DXA and the anthropometric predictive equations of Wilmore & Behnke, 1969 [34] and Oliver et al., 2012 [35], whilst unacceptable agreement was observed between DXA and all other anthropometric predictive equations (Durnin & Wormsley, 1974; Slaughter et al. 1988 and Withers et al. 1987; Table 1) [31,32,33]. Similarly, for LM estimations, the Withers et al. 1987 [33] anthropometric predictive equation demonstrated unacceptable agreement vs. DXA (Table 1), with linear regression suggesting that maturity offset modestly predicted LM for all anthropometric predictive equations.

The findings from the present study suggest that when calculating FM via anthropometric predictive equations, only two equations (Wilmore & Behnke, 1969 and Oliver et al. 2012) demonstrated a good level of agreement when compared to DXA-derived values of FM [34,35]. However, inspection of the Bland–Altman scatterplots as part of the analysis suggests that for all predictive anthropometric equations, notable mean bias, with wide limits of agreement, are present, indicating substantial variability when measuring FM (Figure 2). These findings indicate that whilst the anthropometric prediction equations used within this study may reliably measure FM, they lack sufficient validity to provide accurate estimates of DXA-derived values in professional academy soccer players. Our findings are similar to those of Giro et al. [25] and Lozano-Berges et al. [43], who found that the Durnin and Womersley, 1974 [31], Withers et al. 1987 [33], and Slaughter et al. 1988 [32] (as used in the present study) equations did not accurately predict DXA-derived FM% or BF% in futsal and adolescent soccer players, respectively. Interestingly, findings by Tornero-Aguilera et al. [44] suggest that the anthropometric predictive equation used within their study underestimated FM% when compared to DXA in young male football players (DXA FM%: 19.0% ± 3.7% vs. Anthropometry FM%: 12.7% ± 3.7%). Despite the present study reporting absolute values of FM (in kg; Table 1) and Tornero-Aguilera et al. [44] reporting percentages, our findings are in contrast to those of Tornero-Aguilera et al. [44]. Four of the five anthropometric predictive equations used in the present study (Durnin and Womersley, 1974; Slaughter et al. 1988; Wilmore & Behnke, 1969 and Oliver et al., 2012) overestimated FM vs. DXA-derived values (Table 1) [31,32,34,35]. The reasons underpinning the observed differences between studies remain unclear. Speculatively, the equation adopted by Tornero-Aguilera et al. [44] incorporates different measures to calculate FM than those equations used in the present study, which may help explain the differing findings; however, further research is needed to substantiate this hypothesis.

When considering LM, the findings from the present study indicate that four of the five anthropometric predictive equations (Durnin and Womersley, 1974; Slaughter et al. 1988; Wilmore & Behnke, 1969 and Oliver et al. 2012) demonstrated good agreement vs. DXA-derived values of LM, indicating a strong association between methods (Table 2) [31,32,34,35]. However, Bland–Altman inspection suggests that for all of the anthropometric predictive equations, notable mean bias and wide limits of agreement were observed, indicating substantial variability between DXA and the anthropometric predictive equation when measuring LM (Figure 2). Speculatively, alterations in fat tissue distribution and potential errors in measuring FM via skinfold techniques (despite high intra-rater reliability, ICC: 0.998) may have influenced the LM findings, particularly given the high ICC values (Table 2) and wide limits of agreement between methods. The findings in the present study indicate that whilst the anthropometric prediction equations used within this study may reliably measure LM in professional academy soccer players, they lack sufficient validity to provide accurate estimates of DXA-derived values. This supports previous research in elite, male youth soccer populations, which, despite investigating FFM (and not LM as per the present study), reported large biases between estimated FFM via anthropometric predictive equations and DXA-derived FFM values [23,24]. More specifically, the findings of these previous research studies [23,24] suggest that both the Durnin and Womersley, 1974 [31] and Slaughter et al. 1988 [32] equations (as used in the present study) show good sensitivity in tracking FFM changes within these cohorts. These findings are comparable to those of the present study, which reports good levels of agreement within the equations of Durnin and Womersley, 1974 [31], and Slaughter et al. 1988 [32], despite notable mean bias and wide limits of agreement observed in the Bland–Altman analysis (Figure 2).

The observed contribution of maturity offset within the findings of the present study reinforces the role of biological maturity in LM prediction [26]. The absence of maturity offset calculations as part of the included anthropometric predictive equations likely contributed to the present study’s findings, as somatic development has been shown to alter the relationship between subcutaneous fat and LM, meaning that the same sum of skinfolds may correspond to different LM values depending on a player’s biological maturity [28]. Maturity-related changes in subcutaneous fat distribution and body composition, which directly affect the skinfold values entered into prediction equations, further exacerbate this discrepancy [45]. Speculatively, it may be hypothesized that due to anthropometric predictive equations’ failure to account for the influences of biological maturation (limb proportions, changes in muscle cross-sectional area, etc.) on body composition [26], particularly in youth athlete populations, estimation error may not necessarily reflect measurement inaccuracy. This highlights a potential limitation of anthropometric predictive equations: their reliance on chronological age as a proxy for physiological development, and their shortcomings in accommodating intra-group variability during maturation [26]. Applying these anthropometric predictive equations to individuals with differing biological maturity levels may introduce systematic bias, even within narrowly aged cohorts [36], although further research is needed to confirm this. Regardless of the variable(s) of interest, it should be noted that caution is recommended when applying anthropometric predictive equations in elite youth male soccer populations [23]. Therefore, future research may wish to consider the need for population-specific validation of anthropometric predictive equations, but also for maturity-sensitive approaches in the development of these equations in academy soccer. Speculatively, the authors suggest that embedding maturity offset as a variable within future developed anthropometric predictive equations may help with these approaches.

This study supports the findings of previous research regarding commonly used anthropometric predictive equations in academy soccer players [23,24,43,44]; however, this study is not without limitations. Firstly, despite DXA scans appearing to be minimally influenced by food and fluid consumption [46], it should be noted that, although DXA protocols were standardized in the present study, fasted assessments (as recommended by Nana et al. [37]) were not feasible due to training schedules associated with the pre-season period in professional, academy soccer. Similarly, despite DXA being widely deemed a gold-standard method for body composition analyses due to its accuracy and repeatability [6,7,8], DXA manufacturers’ body composition estimation algorithms are not developed from athletic populations—meaning that reference values are compared against “general” cohorts [7]. Therefore, refining algorithms to better reflect the characteristics of athletic populations (including academy soccer players) may increase the resolution and accuracy of future research when looking at the reliability and validity of anthropometric predictive equations. Moreover, the cross-sectional design limits the ability to understand how anthropometric predictive equation accuracy evolves across different stages of maturation over time, highlighting the need for more longitudinal research [36] and whether the reliability and validity of predictive anthropometric predictive equations differ across wider age categories within academy-age athletes. Despite having a good level of statistical power to detect mean differences within the present study, the external validity is limited by several factors including the narrow age, maturity range and sex of participants. The cohort observed was male academy soccer players, and whether the findings of the present study would be comparable in female cohorts remains an area of future investigation. Lastly, although the findings of this study may be of use to sport science practitioners working with academy soccer, the use of players from a single, UK-based professional soccer academy limits generalizability; therefore, the implementation of these findings within soccer academies of differing age ranges, post-PHV phases, maturity offset characteristics and geographical locations may require investigation to ascertain their suitability.

### Practical Applications

The findings of this study highlight the limitations of commonly used skinfold-based prediction equations for accurately estimating FM and LM in professional, academy soccer players. For practitioners working in these environments without access to more advanced technologies (e.g., DXA), selecting appropriate anthropometric predictive equations must be a priority. Our observed findings suggest that the Wilmore & Behnke, 1969 [34] and Oliver et al., 2012 [35] anthropometric predictive equations offer good levels of agreement for both FM and LM in UK-based professional soccer players. However, due to the wide limits of agreement observed between all equations and DXA, practitioners should be cautious when adopting anthropometric predictive equations and avoid using single-time-point estimates to guide individual player development and welfare decisions. Instead, practitioners should consider consistent longitudinal tracking with the same adopted method, while accounting for biological maturity. In the absence of any currently available anthropometric predictive equations that incorporate maturity offset, practitioners may wish to consider manually implementing these calculations as part of monitoring procedures in academy soccer players. Similarly, the inclusion of relative agreement (i.e., tracking players longitudinally) alongside absolute agreement (i.e., accuracy on an individual level) and whether this is warranted when monitoring body composition in academy soccer players may also be a consideration. Such monitoring should be underpinned by developmentally appropriate interventions in soccer-related training, nutrition, and body composition assessment in academy soccer, where developmental heterogeneity may be a consideration.

## Figures and Tables

**Table 1:** Validity of estimated whole-body fat mass via assessed by dual-energy X-ray absorptiometry and predictive anthropometric equations. Mean difference percentages, coefficient of variation percentages, limits of agreement and effect sizes (*ES*) are presented.

Method							DXA vs. DW				DXA vs. Slau				DXA vs. With				DXA vs. WB				DXA vs. Oliv			
	*DXA*	*DW*	*Slau*	*With*	*WB*	*Oliv*	%Diff	CV%	LoA	*ES*	%Diff	CV%	LoA	*ES*	%Diff	CV%	LoA	*ES*	%Diff	CV%	LoA	*ES*	%Diff	CV%	LoA	*ES*
FM (kg)	9.8 ± 2.0	11.4 ± 2.2	12.6 ± 1.8	8.4 ± 2.1	10.7 ± 1.9	10.3 ± 1.8	16.1 ± 7.0	12.6 ± 5.9	3.81 to −0.73	0.78	22.7 ± 10.8	18.6 ± 9.6	5.66 to −0.63	1.50	13.7 ± 9.1	10.8 ± 7.8	1.06 to −3.37	0.70	11.4 ± 7.9	8.8 ± 6.4	3.06 to −1.33	0.47	10.7 ± 5.8	8.1 ± 4.6	2.62 to −1.62	0.27

CV%: coefficient of variation percentage; DW: Durnin & Wormsley (1974) [31] equation; DXA: dual-energy X-ray absorptiometry; *ES*: effect size; FM: fat mass; LoA: limits of agreement; Oliv: Oliver et al. (2012) [35] equation; Slau: Slaughter et al. (1988) [32] equation; WB: Wilmore & Behnke (1969) [34] equation; With: Withers et al. (1987) [33] equation; %diff: percentage difference.

**Table 2:** Validity of estimated whole-body lean mass via assessed by dual-energy X-ray absorptiometry and predictive anthropometric equations. Mean difference percentage, coefficient of variation percentage, limits of agreement and effect sizes (*ES*) are presented.

Method							DXA vs. DW				DXA vs. Slau				DXA vs. With				DXA vs. WB				DXA vs. Oliv			
	*DXA*	*DW*	*Slau*	*With*	*WB*	*Oliv*	%Diff	CV%	LoA	*ES*	%Diff	CV%	LoA	*ES*	%Diff	CV%	LoA	*ES*	%Diff	CV%	LoA	*ES*	%Diff	CV%	LoA	*ES*
LM (kg)	67.9 ± 6.8	70.2 ± 6.7	69.3 ± 6.5	72.6 ± 7.1	70.7 ± 6.8	71.1 ± 6.9	3.41 ± 0.26	2.5 ± 1.1	4.62 to −0.03	0.35	2.07 ± 0.34	1.8 ± 1.2	4.44 to −1.67	0.21	6.86 ± 0.29	4.8 ± 1.3	7.31 to 2.10	0.69	4.16 ± 0.26	2.9 ± 1.3	5.17 to 0.54	0.42	4.67 ± 0.26	2.9 ± 1.3	5.62 to 0.78	0.48

CV%: coefficient of variation percentage; DW: Durnin & Wormsley (1974) [31] equation; DXA: dual-energy X-ray absorptiometry; *ES*: Effect size; LoA: limits of agreement; LM: lean mass; Oliv: Oliver et al. (2012) [35] equation; Slau: Slaughter et al. (1988) [32] equation; WB: Wilmore & Behnke (1969) [34] equation; With: Withers et al. (1987) [33] equation; %diff: percentage difference.

## Data Availability

Due to the sensitive nature of participant information, the data are not publicly available but may be shared upon reasonable request and subject to ethical approval.

## Abbreviations

The following abbreviations are used in this manuscript:

BIA	Bioelectrical Impedance
BM	Body Mass
BMC	Bone Mineral Content
CV	Coefficients of Variation
DXA	Dual-Energy X-ray Absorptiometry
*ES*	Effect Size
FFM	Fat-Free Mass
FM	Fat Mass
ICC	Intra-class Correlation Coefficient
ISAK	International Society for the Advancement of Kinanthropometry
LoA	Limit of Agreement
LM	Lean Mass
LTAD	Long-Term Athletic Development
PHV	Peak Height Velocity
SD	Standard Deviation
SKF	Skinfolds
U18	Under-18
U21	Under-21
